# Phenotypic Plasticity of Common Wasps in an Industrially Polluted Environment in Southwestern Finland

**DOI:** 10.3390/insects12100888

**Published:** 2021-09-30

**Authors:** Oluwatobi Badejo, Oksana Skaldina, Sirpa Peräniemi, Victor Carrasco-Navarro, Jouni Sorvari

**Affiliations:** 1 Department of Environmental and Biological Sciences, University of Eastern Finland, P.O. Box 1627, FI-70211 Kuopio, Finland; oksana.skaldina@uef.fi (O.S.); victor.carrasco.navarro@uef.fi (V.C.-N.); 2 School of Pharmacy, University of Eastern Finland, P.O. Box 1627, FI-70211 Kuopio, Finland; sirpa.peraniemi@uef.fi; 3 Department of Biology, University of Turku, FI-20014 Turku, Finland

**Keywords:** adaptations, melanization, metals, morphology, TEM-EDX, *Vespula*

## Abstract

**Simple Summary:**

Social insects are ecologically and economically important as ecosystem engineers, agricultural pest predators, pollinators, and seed dispersers. Many of the vespid wasps are social insects. Our study species, Common wasp *Vespula vulgaris*, is native to Finland and classified as invasive in some other parts of the world. The Common wasp have conspicuous yellow and black pigmentation. Their functions and activities in the environment expose the species to environmental pollutants and this study assessed the effect of heavy-metals on common wasps collected from the vicinity of a metal smelter in southwestern Finland. The samples collected were analyzed using various methods such as color morph categorization, electron microscopy, metal analysis, and energy dispersive X-ray analysis (EDX). The methods were used to understand the effects of metal pollution on the species and the adaptive response. Our results indicated phenotypic variation between common wasp samples across the pollution gradient and an adaptive melanin encapsulation process.

**Abstract:**

Insects vary in the degree of their adaptability to environmental contamination. Determining the responses with phenotypic plasticity in ecologically important species in polluted environments will ease further conservation and control actions. Here, we investigated morphological characteristics such as body size, body mass, and color of the common wasp *Vespula vulgaris* in an industrially polluted environment, considering different levels of metal pollution, and we studied the localization of contaminants in the guts of wasps. We revealed some differences in morphological characteristics and melanization of wasps collected in habitats with high, moderate, and low levels of pollution. The results indicated that *V. vulgaris* from highly polluted environments had reduced melanin pigmentation on the face but increased melanin pigmentation on the 2nd tergite of the abdomen. In addition, with transmission electron microscopy (TEM) and energy dispersive X-ray analysis (EDX), we found metal particles from the midgut of wasps originating from the polluted environment. Most of the particles were encapsulated with melanin pigment. This finding confirmed that in wasps, ingested metal particles are accumulated in guts and covered by melanin layers. Our data suggest that wasps can tolerate metal contamination but respond phenotypically with modification of their size, coloration, and probably with the directions of the melanin investments (immunity or coloration). Thus, in industrially polluted areas, wasps might probably survive by engaging phenotypic plasticity with no significant or visible impact on the population.

## 1. Introduction

Exposure of organisms to contaminants might cause toxicity, drive behavioral or structural modifications, or even lead to mortality [1,2,3]. Insects are key animals in the trophic web and are easily affected by pollutants in the environments, and they can be active vectors of pollutant and pathogen spread [4]. Social hymenopterans include different species of ants, bees, and wasps with varying functions in the ecosystems. Social wasps (Vespidae) are important insects in nature, acting as pollinators, seed dispersers, and predators of agricultural pests [5,6,7]. Wasps acquire pollutants with food, water, or while foraging for pulp which serves as a building material for their nests [8].

Although many insects are adapted to pollution through structural, physiological, and phenotypic modifications, there are fitness costs for such an adaptive modification that ensures insect survival in polluted environments. For example, mosquitoes (Diptera, Culicidae) adapt to heavy metals with a reduced reproduction rate and an increase in population-doubling time [9,10,11]. Also, in the lesser fruit fly *Drosophila melanogaster*, the genetic transfer of pollution resistance and a higher rate of female survival across generations were identified. However, this affected the level of reproduction in subsequent generations [12]. This resistance was developed through permanent modulation of the insect genome due to continuous exposure to pollutants by copying genes to enable the production of more enzymes that detoxify the pollutant [13,14]. Similarly, [15] identified some degree of complexity in the uptake and potential bioaccumulation of environmental pollutants in selected Coleoptera and Heteroptera species. The authors suggested that long-term contamination can prompt insects to adopt mechanisms that avoid the accumulation of contaminants.

One such mechanism is the ability to detoxify contaminants to prevent bioaccumulation in Coleoptera [16]. Another mechanism deployed for coping with chemical pollution and a foreign invasion by pathogens in insects is cellular encapsulation [17], a process involving the aggregation of hemocytes around foreign particles and a capsule formation. Melanins are important components in the encapsulation process, and that is one of the reasons why highly melanic insect morphs may possess higher immunity to environmental stress [18].

Insects vary in their adaptive capabilities. This can be due to different factors within the environment, as resistance is expected to be developed through continuous exposure and the exposure dose [12]. Specific examples from social wasps are few, and this leaves an information gap that our study aimed to fill. However, in other hymenopterans, an adaptive response to pollution has been reported. For example, ants have shown a high capacity to bioaccumulate heavy metals [19,20] with a subsequent decrease in immunity and body size. Other studies have reported a decreased abundance of dominant ant species and changes in the distribution of species across the pollution gradient [21]. The authors suggested that the community structure and distribution of ant species across pollution gradients depend on other factors peculiar to the environment and not just pollution intensity. Therefore, environmental pollutants interact with other selective drivers within the ecosystem to prompt an adaptive response from insect species.

The morphological response (phenotypic plasticity) to pollution requires studying because such changes can affect other competitive fitness properties of the insect. The study in [22] showed a decreased area of black facial marking in the common wasp *V. vulgaris* associated with pollution. Furthermore, we expect that some coloration traits can be modular and show different directions of responses in different body parts of the common wasp as it was revealed for the red wood ant *Formica rufa* [23]. Studies of other insects have shown different morphological variations in response to environmental pollutants, such as a decrease in body size in aphids and beetles [24,25] and changes in the wings of *Chironomus riparius* [26,27]. In *V. vulgaris*, research interest in phenotypic plasticity and pigmentation is just emerging and little information is available on the adaptive response to environmental stress and pollution with phenotypic traits.

Here, we studied phenotypic characteristics of *V. vulgaris* workers, such as (1) body size (head width and thorax width); (2) body weight, and; (3) color (abdominal morph and melanization area on the face and abdomen) across a polluted environment from high to intermediate and low pollution zones.

## 2. Materials and Methods

### 2.1. Sample Collection

Samples were collected in the Harjavalta Cu–Ni metal smelter area in southwest Finland. Harjavalta is one of the most polluted places in Finland due to emissions from the smelter. However, the emission levels have progressively reduced over the years and today it is moderately polluted compared to similar sites in other countries [28]. A total of 150 (workers) individual specimens of the common wasp *Vespula vulgaris* (Hymenoptera, Vespidae) were collected during one week in early August 2014 using the beer-trap method [29]. An additional sampling campaign was performed in August 2019, when nine workers of *V. vulgaris* were sampled with a sweep-netting technique specifically for conducting microscopy analyses.

**Figure 1:**
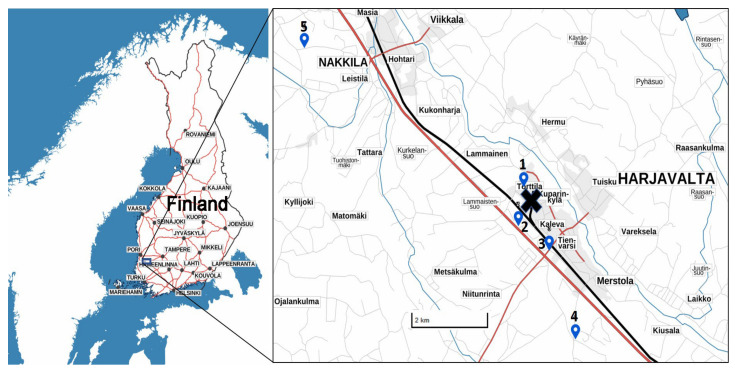
Map showing the location of sample collection. X indicates the position of the smelter, while Torttila (1), and Gate (2) indicate the high-polluted sites. Nummi (3) is the intermediate zone, while Hiite (4), and Nakkila (5) are the reference (low-polluted) zones.

In 2014, wasps were collected from three zones across the pollution gradient (from 0.86 to 10.66 km from the pollution source). Highly polluted sites were named ‘Torttila’ and ‘Gate’ (0.86 km and 0.98 km from the pollution source, respectively). The intermediate zone site was ‘Nummi’, located 1.6 km from the pollution source, and the low-polluted (reference) sites were ‘Hiite’ and ‘Nakkila’, located 4.7 km and 10.6 km from the Harjavalta Cu–Ni metal plant [22] (Figure 1). The sites differed slightly in the general habitat type. Torttila (polluted site) and Hiite (low-polluted site) were old gravel pits partly covered by pines, and the three remaining sites were pine-dominated forests. In 2019, six workers of *V. vulgaris* were captured near the smelter (less than 500 m), and three individuals were captured in the low-polluted areas (more than 8 km away).

### 2.2. Morphological Measurements

Before conducting the quantitative metal analyses, all worker samples were measured for morphological characteristics. Accordingly, wasp faces and thoraxes were individually photographed with a Nikon DS-Fi1 microscope camera (5-megapixel CCD) and a DS Camera Control Unit attached to an Olympus SZX9 trinocular microscope (magnification was ×16). The 8-bit RGB images were taken using NIS-Elements BR imaging software version 3.2. The body size, morphological parameters, and coloration traits were scored using ImageJ and WaspFacer [22] software from the obtained digital images. The body size of *V. vulgaris* workers was measured in head width (HW; the maximum interocular distance) and thorax width (TW; the maximum intertegular distance) [30]. The body weight was measured as the individual dry body mass (M_b_) to the nearest 0.001 mg using a Metler Toledo MX5 analytical balance. After the photographs were taken, all the wasps were dried in a Termaks 8000 oven for 48 h at 60 °C and cooled down for 48 h in a desiccator at 20 °C to prevent additional moisture.

### 2.3. Analyses of Color Variation

**Figure 2:**
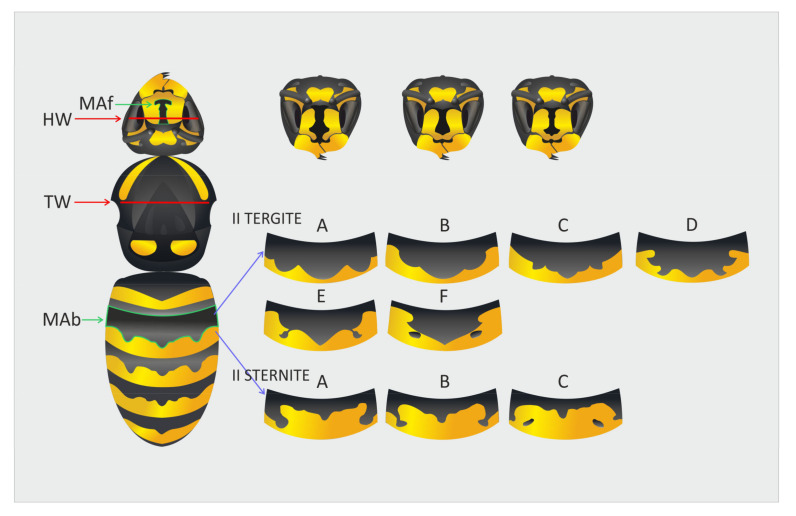
Morphological parameters and color traits scored from the *Vespula vulgaris* bodies: HW (head width), TW (thorax width), MAf (melanization area of the anchor-like melanin facial color trait), MAb (melanization area of the second tergite). Abdominal color morph variations were scored from the second tergite (A, B, C, D, E, F) and second sternite (A, B, C).

Color analyses were performed by assessing head and abdomen coloration traits scored from digital photographs (Figure 2). The coloration traits studied were the melanization area on the face (MAf), melanization area of the abdomen (MAb) and abdomen color morph frequencies. We analyzed the melanization area (MAf) of the wasps’ faces (clypeus) using WaspFacer, recently developed software from [22]. Regarding the wasps’ abdomens, we individually analyzed the color morph frequencies and melanization area on the second tergite and second sternite (the detailed method is described in [31]). The morph categorization followed a modified [32] method described by [31]. In this study, we also added the morph categorization of the sternite of the second abdominal segment of the samples. Within our sample size, six morph categories were identified on the second tergite and three morph categories on the second sternite (Figure 2). The melanization area of the 2nd tergite of the abdomen (MAb) was measured as the total melanized area in mm^2^ using a free tool in ImageJ software.

### 2.4. Metal Analyses

To quantify metal elements from *V. vulgaris* workers, we used an inductively coupled plasma mass spectrometer (ICP-MS). The method enabled the identification of the following elements: arsenic (*As*), cadmium (*Cd*), cobalt (*Co*), copper (*Cu*), iron (*Fe*), mercury (*Hg*), nickel (*Ni*), lead (*Pb*), and zinc (*Zn*). The instrument was calibrated using a certified solution standard TraceCERT Periodic Table Mix 1 and Mercury standards for ICP, Sigma Aldrich. Element isotopes without known spectral interferences were preferentially selected for analysis. Polyatomic interferences were removed using a triple-quadrupole reaction system for elements that do not have abundant, interference-free isotopes. The reaction system was operated in collision mode with kinetic energy discrimination (KED), using helium as the cell gas (3.7 mL min^−1^). A sample injection was performed with a peristaltic pump and nebulizer. The analyses included three internal standards: (1) lithium-6, (2) rhodium-103, and (3) uranium-238. They were mixed online with the samples to compensate for matrix effects and instrument drift. The blanks and certified standard reference material (NIST^®^ SRM^®^ 1577b Bovine Liver Sigma-Aldrich, Saint Louis, MO, USA) were included. They were additionally measured with the unknown samples for each batch of analysis, aiming to check for contamination and to confirm the accuracy of the analysis batch. The recovery rate for different elements in standard was >95%.

Individual wasp samples (0.015–0.020 g) were dissolved using a microwave digestion system (MARS^TM^ 6 iWave instrument CEM Corporation, Matthews, NC, USA) and the Animal Tissue method in 8 mL of HNO_3_ (TraceMetal^TM^ grade, Fisher Scientific, Waltham, MA, USA) using MARSXpress Teflon digestion vessels. The use of both analytical blanks and certified reference material allowed us to confirm that the accuracy of the method was within acceptable limits. After digestion, the samples were diluted to 20 mL with de-ionized water (USF Elga Maxima, Woodridge, IL, USA). The determination of metal concentrations was performed with a NexION 350D ICP-MS (PerkinElmer, Waltham, MA, USA) equipped with an ESI prepFAST autosampler (Elemental Scientific, USA). Each metal element was detected at the approximately 0.0001 µg/g detection limit.

### 2.5. Transmission Electron Microscopy (TEM) and Energy-Dispersive X-ray Analyses (EDX)

Nine wasp specimens were collected alive using the hand-net insect-collection method. Six individuals were trapped in areas with high pollution levels, and three samples in the areas, where pollution was low. After approximately 2 h, their abdomens were dissected and placed in a Karnovsky fixative solution: glutaraldehyde (1.5%) mixed with paraformaldehyde (2%) in a Sörensen phosphate buffer 0.1 M, pH 7.4. Samples were stored in a fixative solution for approximately three weeks. After that, the guts were separated from the other body tissues, and the midguts were dissected for further analyses. For the lightning microscopy (LM) and transmission electron microscopy (TEM), a post-fixation process was performed to improve the localization of metals in the tissue. Post-fixation was made in 1% sodium sulphide (Na_2_S in 0.1 M phosphate buffer (pH 7.4) for 2 h. The samples that went to energy-dispersive X-ray analyses (EDX) were not treated with post-fixatives. Further, washing in 0.1 M phosphate buffer (pH 7.4) was performed, followed by dehydration of the alcohol series (10 min each)—the concentrations used were 50%, 70%, 90%, 94% (one time), and 100% (three times)—followed by dehydration with propylene oxide for 15 min and then 10 min. Those manipulations were performed under stable room-temperature conditions. Further, infiltration was performed with a propylene oxide and LX-112 resin (epon) mixture (1:1) for 2 h, and then with pure resin (epon) overnight. Embedding was performed with pure resin (epon). Polymerization was conducted in an oven at +60 °C for 48–72 h. Sections were obtained using ultramicrotome Leica EM UC7. Thereafter, thin sections (1 µm) were taken under a light microscope and stained with toluidine blue. After selecting the areas of interest, thin sections for TEM (about 70 nm) were cut and used for TEM analyses without staining. LM was performed prior to TEM to select the specific areas of potential metal accumulation in gut tissues. After pre-processing, three samples trapped in polluted areas and two reference samples, were suitable for further analyses. Only the three samples from the polluted zones were used for the EDX analysis.

Analysis was performed in SIB Labs (University of Eastern Finland) by using high-resolution transmission electron microscope JEM-2100 F (Jeol Co., Tokyo, Japan) connected to digital camera Quemesa 11MPix. Results were further analyzed using Pathfinder 1.4 software.

### 2.6. Statistical Methods

All statistical analyses were carried out using SAS 9.4 statistical software (SAS Institute, Inc.). Differences in heavy-metal concentrations, heavy-metal loads (unrotated principal components), and body-size measures between pollution zones were analyzed using restricted maximum likelihood based linear mixed models using trap identity as a random variable and the Kenward–Roger approximation method for the degrees of freedom. The association between metal levels and different color morphs of second gastral tergites were analyzed using similar linear mixed models. Metal concentrations were log10 transformed prior to analyses. Model estimates (estimated marginal means and confidence intervals, CI) for log10-transformed values were back-transformed to the original scale for tables and figures. Homogeneity of variances was detected visually, and unequal variances were controlled using SAS code for the unequal variances mixed model (REPEATED/GROUP = zone). The similarity of morph frequencies was analyzed with likelihood ratio Chi-square tests. Normality of residual distribution and variance homogeneity in mixed model analyses were inspected visually. Correlations were made using Pearson’s correlations.

## 3. Results

### 3.1. Metal Concentrations in Wasps

Concentrations of *Hg* were detected only in the two high-polluted sites, and the levels did not differ between those sites (Torttila: 0.087 ± 0.026 µg g^−1^; Gate: 0.076 ± 0.013 µg g^−1^; *F*_1,60_ = 0.62, *p* = 0.43). Of the other metals, *Co*, *Ni*, *Cu*, *As*, *Cd*, and *Pb* differed between zones, whereas *Fe* and *Zn* did not (Table 1). The concentrations of metals other than *Fe* and *Zn* were highest in the polluted zone and lowest in the reference zone.

**Figure 3:**
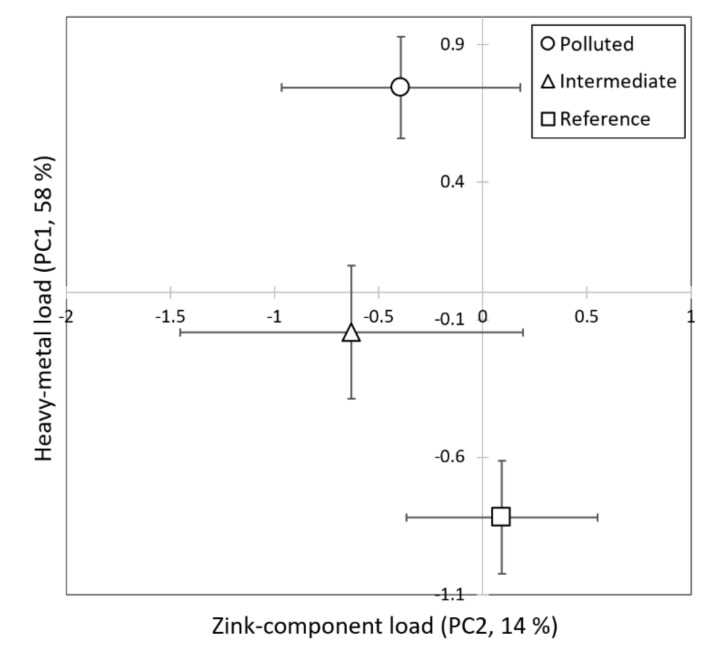
Mean heavy-metal load factors (PC1 and PC2 ± 95% CI) in the pollution zones. All zones differed significantly from each other in PC1 but not in PC2.

Principal component (PC) analysis was carried out without the levels of *Hg* since it was below the detection-limit levels in the intermediate and low-polluted zones. The principal component analysis resulted in two principal components that had eigenvalues above 1 (PC1: 4.68, PC2: 1.15). PC1 was strongly or moderately correlated with all the metals except *Zn* (correlations over 0.6; Table 2), and PC2 was strongly correlated with Zn (correlation 0.91) (Table 2). The combined metal burden (PC1) was highest in the high-polluted zone and lowest in the low-polluted zone, whereas the Zn component burden (PC2) did not differ among zones (PC1: *F*_2,145_ = 63.46, *p* < 0.0001; PC2: *F*_2,11.6_ = 1.87, *p* = 0.20; Figure 3).

### 3.2. Body Size & Weight

A between-zone comparison revealed that the yellowjackets significantly differed in TW but not in HW and M_b_ (TW: *F*_2,145_ = 4.91, *p* = 0.0003; HW: *F*_2,8.88_ = 3.10, *p* = 0.095; M_b_: *F*_2,145_ = 2.87, *p* = 0.89). Thoraxes were widest in the high-polluted zone and narrowest in the low-polluted zone (mean ± 95% CI, polluted: 2.49 mm ± 0.03; intermediate 2.46 mm ± 0.04; reference 2.39 mm ± 0.03; pairwise Tukey tests: polluted vs. intermediate *p* = 0.18, polluted vs. reference *p* < 0.0001, intermediate vs. reference *p* = 0.025). Correlation analyses revealed that there was a weak positive correlation between HW and ITD (*r* = 0.232, *p* = 0.0046).

### 3.3. Color

HW had a weak negative correlation with the size of the clypeus melanization area and a weak positive correlation with the second abdominal tergite melanization area (MAf: *r* = −0.245, *p* = 0.0027; MAb: *r* = 0.232, *p* = 0.0064). This suggested that *V. vulgaris* samples with smaller heads had slightly larger facial melanin markings and smaller abdominal tergite melanin marking. ITD had no correlation with MAf and a weak positive correlation with MAb (MAf: *r* = −0.089, *p* = 0.279; MAb: *r* = 0.185, *p* = 0.031). We revealed a negative correlation between M_b_ and MAf (*r* = −0.273, *p* = 0.0012).

**Figure 4:**
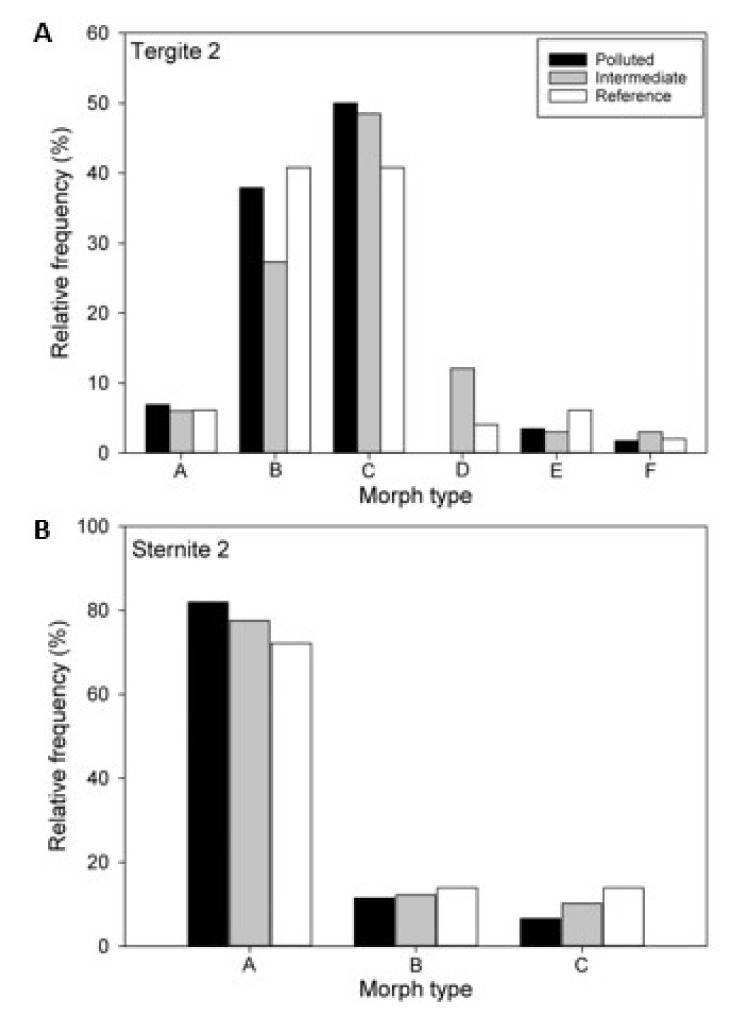
Relative frequencies of different color morphs of 2nd tergites (**A**) and 2nd sternites (**B**) of common wasps in three pollution zones.

Morph frequencies of both the second tergite and second sternite did not differ between high-polluted, intermediate, and low-polluted zones (likelihood ratio tests, Tergite 2: df = 10, *G*^2^ = 10.542, *p* = 0.39; Sternite 2: df = 4, *G*^2^ = 1.672, *p* = 0.80; Figure 4). Tergite 2 morphs did not differ in their heavy-metal burdens (PC1: *F*_5,121_ = 0.32, *p* = 0.90; PC2: *F*_5,125_ = 0.12, *p* = 0.99). Similarly, there were no differences in heavy-metal burdens between Sternite 2 morphs (PC1: *F*_5,130_ = 1.52, *p* = 0.22; PC2: *F*_5,134_ = 0.09, *p* = 0.91).

**Figure 5:**
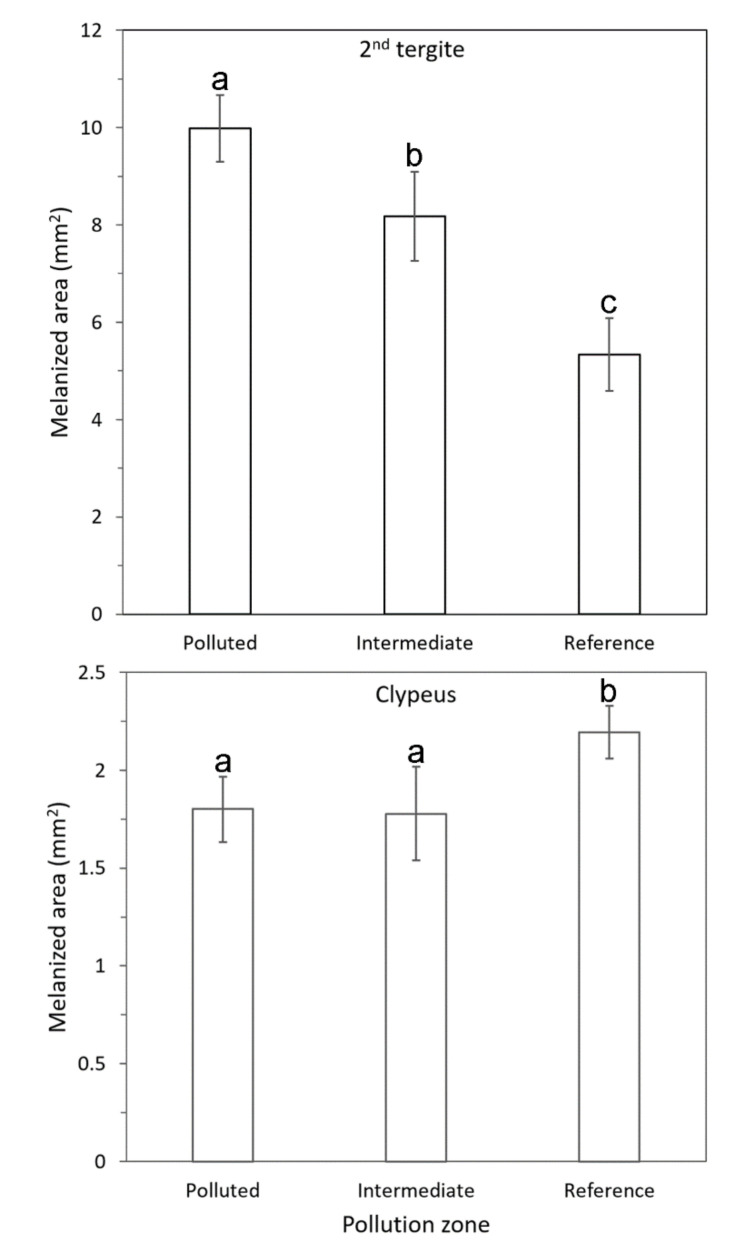
Mean size (±95% CI) of the melanized marking in the clypeus and second tergite in the three pollution zones. A different letter above the bars indicates a significant pairwise difference (Tukey’s test *p* < 0.05).

The melanized marking on the clypeus, MAf, differed significantly among zones (*F*_2,11.4_ = 10.24, *p* = 0.0029). Wasps from the low-polluted zone had a significantly larger MAf than those from the Intermediate and Polluted zones (Figure 5). The MAf decreased with increasing heavy-metal burden PC1, whereas it was not associated with the zinc burden PC2 (PC1: *r* = −0.314, *p* = 0.0001; PC2: *r* = 0.060, *p* = 0.47). Furthermore, the melanized marking on the second abdominal tergite, MAb, differed among the zones (*F*_2,136_ = 41.44, *p* < 0.0001). However, the association between MAb and the zones was opposite to that of the MAf (Figure 4). The MAb increased with an increase in the heavy-metal burden PC1, whereas it was not associated with the zinc burden PC2 (PC1: *r* = 0.322, *p* = 0.0001; PC2: *r* = 0.141, *p* = 0.10). There was a weak negative correlation between MAf and MAb (N = 137, *r* = −0.273, *p* = 0.0012).

### 3.4. Transmission Electron Microscopy (TEM) and Energy-Dispersive X-ray Analyses (EDX) Analyses

**Figure 6:**
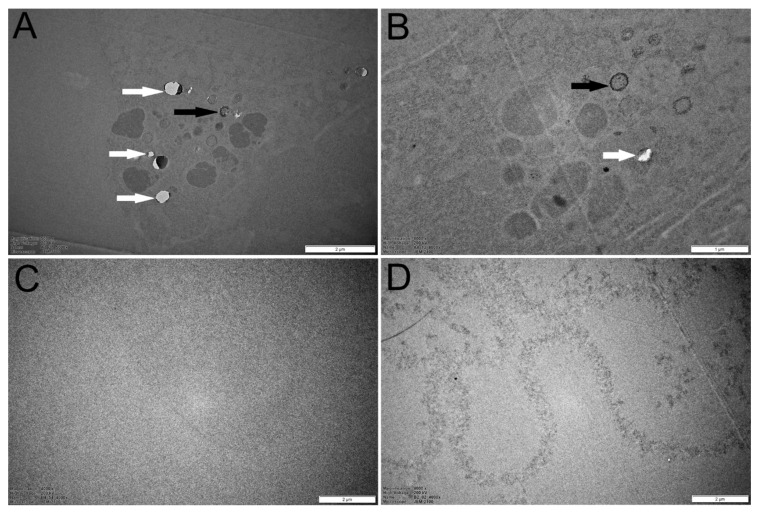
TEM images captured from the midgut tissues from the *Vespula vulgaris* workers captured in the area close to the smelter classified as the polluted zone (**A**,**B**) and Nakkila area classified as the reference zone (**C**,**D**) in 2019. The white arrows show the shiny metal particles revealed with the EDX technique. The black arrows show particles already encapsulated with melanin pigment. The quality of the images was affected by the inability to stain samples because of the EDX analysis.

TEM imaging revealed that *V. vulgaris* samples collected in high-polluted environments contained black encapsulated granules in their midguts’ cellular tissues, while no such granules were revealed in individuals from control habitats (Figure 6). The EDX analyses revealed that those particles were associated with heavy metals such as *Al*, *Co*, *Cu*, *Mn*, *Cr*, *Fe*, and *Ni*. The elevated level of *Cu* in the EDX spectra originated from the TEM grid and was not indicative of the level of *Cu* pollution (Supplementary Figure S1). In one individual, we have found a granule with *Hg*. These results suggest that, in polluted environments, wasps are exposed to high amounts of heavy metals, which are further accumulated in their guts as encapsulated granules.

## 4. Discussion

Phenotypic plasticity has been shown to have direct links to different drivers within the environment, most importantly, temperature and genetic drivers [33,34]. Phenotypic plasticity is mostly expressed as morphological differences within individuals of a species, referred to as morphs. Morphs have been grouped based on wing morphology [35,36,37], coloration [31,32], and body size [38]. Other important factors that can drive morph selection is the condition of growth. Insects tend to invest more in morphs that can survive better in the environment. Environmental pollution can alter the condition of growth in insect species and affect the phenotypic properties of emerging adults. These developed properties provide a competitive advantage for individual insects within the population. Our study area had considerable metal contamination, and we assessed the collected samples to determine the variation in morphs, but we discovered there was no significant difference in morph distribution across the pollution gradient. This is possibly because this species may have the capacity to build up an immune response when continuously exposed to contaminants, or the melanin production was not limited by metal pollutants in the environment.

The concentration of metals accumulated in insects depends on the level of contamination of the environment and the feeding habit of the insect. Therefore, we expect the concentration from our samples to be peculiar to the exposure level in the location. The concentrations of heavy metal in our samples are similar to those reported in other insect species; e.g., in carabid beetles, the levels of *Cd*, *Fe*, and *Pb* were within a similar range [39] as our results, while *Cu* and *Zn* concentrations from our study exceeded those reported for the beetles. In a closer vespid relative *Polistes nimphus* wasps, bioaccumulation of *Zn* was higher than our results while *Cd* and *Pb* were lower [40]. Similarly, in *P. dominulus*, *Pb* concentration was lower than the concentration from our result, following similar trends from the polluted to the reference zone [41]. Further, in a recent study in *Vespa velutina* and *Vespa crabro*, the reported concentrations of *Pb* and *Fe* were also much lower, presumably due to low environmental exposure [42]. *P. dominulus* showed evidence of effective regulation of metal contaminant while in carabid beetles, [43] reported histological and ultrastructural anomalies in the midgut due to heavy-metal accumulation, but similar anomalies were not observed in common wasps.

We found that melanin pigments in the midgut epithelium tissue formed capsules or granules around ingested metal contaminants. These granules were associated with specific metal elements such as *Al*, *Co, Mn*, *Cr*, *Fe*, and *Ni*. The formation of capsules abundant in metals as a storage mechanism is common in invertebrates [44,45]. These capsules are often located in the digestive epithelium or tissues that surround it, and serve to sequester metals, therefore acting as a detoxification mechanism. However, the accumulation of metals in these encapsulations could be another vector of exposure for metals to upper trophic levels, although their availability is not clear and may depend on many factors such as the specific metals and prey [26,46].

The midgut is an important location in the insect immune defense because it is the point of digestion and absorption of nutrients, and the epithelium provides a suitable point of penetration of foreign pathogens into the body tissue. Additionally, the midgut extends through the entire length of the abdomen and provides a considerable surface area for penetration. Furthermore, the foregut and the hindgut are protected with chitinous material, thus making the midgut a possible point of pathogen attack in the digestive tract. Although the midgut has a chemical mechanism for killing pathogens, inorganic pollutants are effectively immobilized by melanization and subsequent encapsulation of the pollutants. The melanization process starts with the conversion of tyrosine to melanin precursors in the presence of phenoloxidase. The precursors are crosslinked to hemolymph protein to form a layer of melanin that surrounds and sequesters invading pollutants and pathogens [47]. Therefore, we consider that the wasps in the polluted zone deployed the melanin pigments in immobilizing the pollutants and hindering absorption into body tissues. It is plausible that the encapsulation process of metal pollutants in wasps from the polluted zone was responsible for the reduction in melanin pigmentation on the face because wasps from the reference zone had larger melanin pigmentation on their clypeus (MAf) and the heavy-metal burden PC1 in the polluted zone correlated directly with the reduction in clypeus melanization.

Evidence of modularity in the allocation of melanin pigmentation was observed as an opposite trend in the pigmentation on the clypeus and the tergites. This suggests that parts of the body of the common wasps might have varying response to environmental contaminants. Modularity in the expression of melanin pigmentation might affect the functions of pigments [48] in wasps and other social insect species.

The morphological differences observed across zones were restricted to the clypeus and the thorax, while there was no significant difference in overall growth (dry body mass) and abdominal pigmentation. Therefore, morph selection and adaptation in the common wasp is nonlinear. Our previous studies [31,49] have shown high variability in cuticular melanization of the abdomen in response to climate and temperature. This study has shown that pigmentation of different body parts of the common wasps can respond differently to selective pressures within the environment. Although the importance of melanin as an important pigment for immunity and morph selection is established, facial markings might provide more information on the adaptive strength of the common wasp.

## 5. Conclusions

There was variation in melanic pigmentation on the clypeus and abdominal tergites across the pollution gradient. The variation in melanin pigmentation on the clypeus corresponded with the level of pollution across the zones, while growth parameters were constant. Metal particles were identified in the midgut of wasps from the polluted zones. These particles were encapsulated by melanin pigments, and absorption into body tissue was prevented. Therefore, common wasps *Vespula vulgaris* can survive within a polluted environment by deploying melanin for immune response purposes. However, the level of melanic immune response might not be sufficient for proper physiological activities and growth of the common wasp in more-polluted environments. Therefore, environmental contamination and pollution should be prevented to ensure sustenance of ecologically important species such as the common wasp *V. vulgaris*.

## Figures and Tables

**Table 1:** The average heavy-metal concentrations (µg g^−1^ ± 95% confidence interval) of *Vespula vulgaris* specimens in the three studied pollution zones and the results of linear mixed models. The same letter symbol for the metal levels denotes a statistical pairwise similarity between sites (Tukey’s test *p* > 0.05).

	Polluted Zone (N = 62)	Intermediate Zone (N = 36)	Reference Zone (N = 52)	Test Result
*Fe*	233.18 ± 1.20	202.63 ± 1.19	186.77 ± 1.10	*F*_2,6.75_ = 2.85, *p* = 0.13
*Co*	1.41 ± 1.25 ^a^	0.51 ± 1.29 ^b^	0.39 ± 1.26 ^b^	*F*_2,88.9_ = 35.39, *p* < 0.0001
*Ni*	11.25 ± 1.78 ^a^	9.21 ± 3.67 ^a^	3.43 ± 1.54 ^b^	*F*_2,3.72_ = 9.61, *p* = 0.034
*Cu*	84.24 ± 1.23 ^a^	56.34 ± 1.25 ^b^	46.05 ± 1.13 ^b^	*F*_2,6.49_ = 16.82, *p* = 0.0027
*Zn*	544.63 ± 1.61	352.45 ± 1.83	531.74 ± 1.35	*F*_2,6.54_ = 1.52, *p* = 0.29
*As*	6.98 ± 1.45 ^a^	3.97 ± 1.73 ^a^	1.63 ± 1.33 ^b^	*F*_2,6.53_ = 25.60, *p* = 0.0008
*Cd*	1.60 ± 1.29 ^a^	0.41 ± 1.28 ^b^	0.34 ± 1.22 ^b^	*F*_2,89.4_ = 50.72, *p* < 0.0001
*Pb*	1.26 ± 1.81 ^a^	0.82 ± 2.42 ^a^	0.20 ± 1.60 ^b^	*F*_2,8.87_ = 16.05, *p* = 0.0011

**Table 2:** Correlations between logarithmically transformed metal eigenvalues and two principal components (PC1 and PC2).

	Factor Pattern	
	PC1	PC2
*Fe*	0.615	−0.221
*Co*	0.840	0.317
*Ni*	0.722	−0.380
*Cu*	0.916	−0.087
*Zn*	0.303	0.907
*As*	0.845	−0.153
*Cd*	0.859	0.076
*Pb*	0.831	0.016

## Data Availability

The data presented in this study are available on request from the corresponding author.

## Supplementary Materials

The following are available online at https://www.mdpi.com/article/10.3390/insects12100888/s1, Figure S1: EDX spectrum for three samples (individuals) of *V. vulgaris* guts captured in polluted environments in vicinity of Harjavalta Cu–Ni smelter. Letters A–C indicate the wasps’ ID numbers.
