# Supplementary material for: Phenotypic Plasticity of Common Wasps in an Industrially Polluted Environment in Southwestern Finland
Source: Insects. 2021 Sep 30;12(10):888. doi: 10.3390/insects12100888 (PMC8540993; doi:10.3390/insects12100888)
Supplement: Supplementary file 1 [file insects-1380677-supplementary.pdf › insects-1380677-supplementary.pdf]

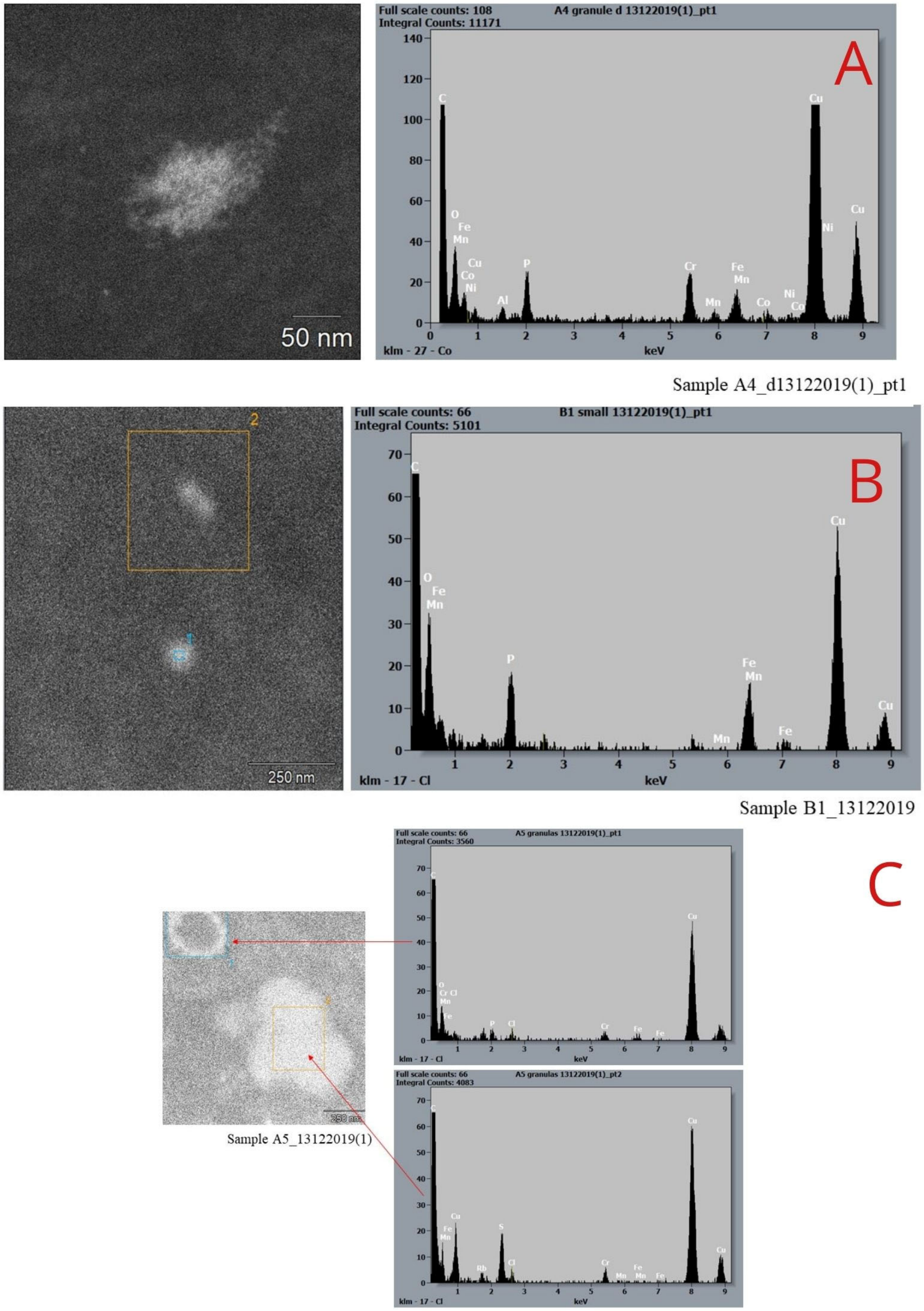

Figure S1. EDX spectrum for three samples (individuals) of V. vulgaris guts captured in polluted environments in vicinity of Harjavalta Cu–Ni smelter. Letters A–C indicate the wasps' ID num-bers.
